# Arterial tissue transcriptional profiles associate with tissue remodeling and cardiovascular phenotype in children with end-stage kidney disease

**DOI:** 10.1038/s41598-019-46805-5

**Published:** 2019-07-16

**Authors:** Christian Freise, Betti Schaefer, Maria Bartosova, Aysun Bayazit, Ulrike Bauer, Thomas Pickardt, Felix Berger, Lars Melholt Rasmussen, Pia Søndergaard Jensen, Guido Laube, Francesca Mencarelli, Klaus Arbeiter, Rainer Büscher, Sandra Habbig, Kristina Möller, Marietta Kirchner, Franz Schaefer, Claus Peter Schmitt, Uwe Querfeld

**Affiliations:** 1 Department of Pediatric Nephrology and Center for Cardiovascular Research, Charité - University Medicine, Berlin, Germany; 2 Division of Pediatric Nephrology, Center for Pediatric and Adolescent Medicine, University of Heidelberg, Heidelberg, Germany; 3 Division of Pediatric Nephrology, Cukurova University, School of Medicine, Adana, Turkey; 4 The National Register for Congenital Heart Defects, Berlin, Germany; 5 Department of Clinical Biochemistry and Pharmacology, Center for Individualised Medicine in Arterial Diseases (CIMA), Odense University Hospital, Odense, Denmark; 6 Department of Nephrology, Kinderspital Zürich – Eleonorenstiftung, Zürich, Switzerland; 7 Department of Pediatrics, S. Orsola-Malpighi Hospital, Bologna, Italy; 8 Pediatric Nephrology, University Children’s Hospital, Vienna, Austria; 9 Clinic for Pediatrics II, Essen University Hospital, Essen, Germany; 10 Department of Pediatric Nephrology, University Children’s Hospital, Cologne, Germany; 11 Department of Pediatrics, Klinikum Links der Weser, Bremen, Germany; 12 Institute of Medical Biometry and Informatics, University of Heidelberg, Heidelberg, (M.K.) Germany

**Keywords:** Paediatric research, End-stage renal disease

## Abstract

Chronic kidney disease (CKD) greatly increases the risk for cardiovascular disease (CVD). However, molecular mechanisms underlying CKD-induced arterial remodeling are largely unknown. We performed a systematic analysis of arterial biopsies from children with stage 5 predialysis CKD participating in the Cardiovascular Comorbidity in Children with Chronic Kidney Disease (4 C) study. For comparison, we studied biopsies from children without CKD, coronary bypass vessels from adults with atherosclerotic coronary heart disease without CKD and aortic sections of subtotally nephrectomized rats. In pediatric CKD patients, gene expression was correlated to the cardiovascular phenotype assessed by surrogate end-points. The arterial calcium content correlated with the intima-media thickness (IMT) of biopsied vessels from pediatric CKD patients, was markedly increased compared to biopsies from children without CKD and comparable to adult coronary bypass patients. Significant transcriptional changes included ECM components, pro-calcifying factors, and physiological calcification inhibitors; most were highly accordant with changes observed in adults with atherosclerosis and in uremic rats. Individual gene expression levels were significantly associated with the left ventricular mass index and carotid intima media thickness. Thus, inflammatory processes (TNF, IL-10), calcification inhibitors (CA2), the Wnt-pathway (FGF-2) and foremost, ECM components (HMGA1, VNN1, VCAN), impact pathobiological responses in arteries from children with CKD.

## Introduction

Chronic kidney disease (CKD) is an independent risk factor for cardiovascular disease (CVD) and greatly increases the risk for cardiovascular events in patients of all ages. The arterial system is particularly affected by CKD as evidenced by the frequent development of vascular calcifications, which are observed even in children^1–3^. Vascular calcifications are promoted by a plethora of risk factors occurring in CKD, but are considered late events in a process of vascular remodeling that seems to involve the entire arterial system^4–6^. In contrast, little is known about the earliest stages of CKD-associated systemic vascular disease.

Pediatric patients with CKD comprise only a small fraction of the total CKD population, but appear uniquely suited to study the effects of “native CKD” on the cardiovascular system due to the virtual absence of vascular morbidity related to ageing, diabetes and life-style related factors such as smoking. In an ongoing prospective observational study, the Cardiovascular Comorbidity in Children with CKD (4 C) study, a consortium of pediatric nephrologists in Europe has joined to investigate the evolution of CVD of children as they advance through successive stages of CKD^7,8^.

We have performed an *ex-vivo* study of arterial biopsies obtained prior to dialysis initiation or transplantation from children participating in the 4 C study who had progressed to end-stage kidney disease (ESKD). The aim of the study was to characterize the origins of CKD-related arterial disease in this unique population.

Arterial biopsies were analyzed for morphological abnormalities, calcium contents and gene transcription levels (customized PCR array for 88 selected genes). To specify CKD-associated changes, results were compared to arterial biopsies from children undergoing vascular surgery and to biopsies taken from adults with coronary heart disease. To further assess the generalizability of the transcriptional changes observed in the CKD specimens, we compared the human findings with those obtained in arteries of rats with subtotal nephrectomy, a standard rodent CKD model. Moreover, we explored potential associations of the tissue level findings with the clinical phenotype as characterized by surrogate markers of CVD, measured at baseline and during follow-up of the 4 C study.

## Methods

### Study populations

Children aged 6–17 years with a GFR of 10–60 ml/min/1.73 m^2^ (calculated at the time of enrollment using a cystatin C/creatinine-based formula^9^) not yet receiving renal replacement therapy were eligible for the ongoing observational 4 C study, which is prospectively following 688 pediatric patients with CKD at 55 pediatric nephrology centers in 12 European countries (Clin Trials.gov NCT01046448). All patients undergo annual cardiovascular assessments including carotid ultrasound, PWV measurement, echocardiography and 24-hour ambulatory BP monitoring.

The study was approved by the Ethics Committee of the Medical Faculty of the University of Heidelberg (S-032/2009) and by local ethics committees in all participating centers, and parents or legal guardians provided informed written consent for study participation including permission of an arterial biopsy^7^. In a subproject of the 4 C study, patients with ESKD (but without prior renal replacement therapy) undergoing first-time kidney transplantation, Tenckhoff catheter insertion for peritoneal dialysis or fistula creation for hemodialysis and/or their guardians were asked to donate pieces of arterial tissue removed during the procedure for the study. All methods were performed in accordance with the relevant institutional guidelines and regulations.

### Control populations

#### Children

Arterial control biopsies from children without CKD were obtained from the biobank of the National Register for Congenital Heart Defects, a national repository for medical data on patients with congenital heart defects in Germany. The collection of samples and provision for research studies is approved by the ethics committee of the Berlin Charité Medical Faculty (EA2/131/10) and informed written consent of patients and parents.

#### Adults

Control biopsies from adults without CKD were obtained from the Centre for Individualized Medicine in Arterial Diseases (CIMA) in Odense, Denmark. Repair arteries from coronary bypass surgery (internal thoracic artery) were collected at the Department of Thoracic, Heart and Vascular Surgery, Odense University Hospital, Denmark consecutively from 2008 to 2012. All participants gave written informed consent, and the study was approved by the Ethical Committee of Region Southern Denmark (S-20100044). Immediately after surgery, the internal thoracic artery was dissected free from the surrounding tissue. An arterial ring was cut and short term formalin-fixed (approximately 24 h in 4% buffered paraformaldehyde), then moved to a PBS solution and subsequently embedded in paraffin.

#### Rat aortas

Aortas from subtotally nephrectomized rats (n = 10) were obtained from a previous study^10^ and were processed like the human biopsies, including their application in a rat customized RT^2^ Profiler PCR Array (see below). All experimental protocols were approved by institutional review boards and state health authorities (LAGeSo; G0019/11). All animal experiments were conducted in accordance with local institutional guidelines for the care and use of laboratory animals.

### Processing of arterial biopsies

Arterial biopsies were collected according to a standardized protocol. Biopsied vessels were gently cleaned of surrounding fat tissue, immediately cut up into 3 pieces and placed into vials containing 4% buffered formalin or RNAlater; the remaining tissue was immediately deep frozen in liquid nitrogen. Formalin-fixed samples were later embedded in paraffin, and deep frozen samples stored at −80 °C; all samples were sent to the study center in Berlin for further processing.

Paraffin embedded biopsies were investigated by histochemistry. The morphology of the biopsies was assessed using hematoxylin and eosin (H/E)-stained tissue sections. The H/E-stained sections were also used to determine the intima-media thickness (IMT). Using the Image J program 1.42q (National Institutes of Health, Bethesda, MD, USA), the distance from the lumen/intima edge to the media/adventitia edge of the arteries was measured at 12 different spots per arterial section and the average expressed as IMT (mean ± SD). The extent of media calcifications was quantified by *von Kossa*- and alizarin red-staining and calcification positive areas were quantified using Image J software and expressed as percentage of total arterial wall area.

Cryoconserved biopsies were divided into two segments using a scissor. One part was used to determine the whole calcium content of the biopsies, the other part was used to isolate mRNA for gene expression studies.

### Determination of calcium content in biopsies

The cryoconserved biopsy segments were washed in calcium- and magnesium-free phosphate buffered saline and dried on Kimwipes® disposable wipers (Sigma-Aldrich, Deisenhofen, Germany). The segments were subsequently weighed and mixed with 100 µl 0.1 mol/l HCl in a 1.5 ml reaction tube. After 18 h incubation at 20 °C, the tubes were vortexed and centrifuged for 30 s at 10.000 × g. Calcium contents in the supernatants were determined by cresolphthalein complexone chemistry (OCPC).

In parallel, the respective protein contents in the tissues were determined in the remaining sediments. After removing the HCl supernatants, the sediments were washed with demineralized water before 300 µl 0.1 mol/l NaOH/1% SDS were added. The mixture was homogenized by ultrasound, vortexed and then incubated for 1 h at 20 °C. Afterwards, the probes were vortexed for 30 s at 10.000 × g and protein contents in the supernatants were determined using a BCA-kit (Thermo Fisher Scientific, Waltham, MA, USA).

The calcium content of each probe was finally normalized to the weight and to the protein content.

### Customized PCR-Array

Only probes with sufficient tissue mass and with an unbroken cold chain were used for RNA isolation. A sufficient size of the probes was determined by a skillful molecular biologist on the basis of past experience. RNA was isolated using the Trizol reagent (Invitrogen, Carlsbad, California, USA) and phenol chloroform extraction. RNA quantity was estimated by a NanoDrop ND-1000 device (NanoDrop Technologies, Wilmington, NC). Isolated RNA with a ratio of absorbance at 260 nm and 280 nm of < 2 was not applied to the PCR array. Gene expressions in human and rat vessel biopsies were determined using a human/a rat customized RT^2^ Profiler PCR Array (Qiagen, Hilden, Germany). For the customized PCR array, all genes of interest were preselected by the authors. 500 ng RNA per probe were transcribed into cDNA using the RT^2^ First Strand Kit (Qiagen). The cDNA was mixed with the RT^2^ SYBR Green Master mix (Qiagen), loaded on the PCR array plate and the PCR was run on a MX3005 system (Agilent Technologies, Santa Clara, CA, USA).

The customized RT^2^ Profiler PCR Array plates (Qiagen) contained primers for 93 preselected genes including five housekeeping genes^11^ and internal controls. The acronyms and the respective full names of all genes are given in Supplementary Table S1. All expression data were calculated using the ΔΔCT method. Gene expressions in biopsies were normalized to the mean gene expressions of five housekeeping genes: HPRT1 (Hypoxanthine Phosphoribosyltransferase 1), PGK1 (Phosphoglycerate Kinase 1), EIF2B1 (Eukaryotic Translation Initiation Factor 2B Subunit Alpha), PPIA (Peptidylprolyl Isomerase A), and ELF1 (E74 Like ETS Transcription Factor 1). Relative gene expressions in the 4 C biopsies were determined by computing the ratio between the 4 C biopsies and the biopsies from the respective control populations.

To generalize the results of the gene expression studies, the preselected genes were clustered in different functional categories such as extracellular matrix proteins or ion channels. This was done according to published evidence (see also the review by Rutsch *et al*.^12^) allowing a classification of the different genes.

### Correlation studies

Vascular measurements performed at baseline and annually during follow-up in the 4 C study included echocardiography, 24-hour ambulatory blood pressure monitoring (ABPM), carotid ultrasound, and pulse wave velocity^8^. All examinations were performed in a standardized manner according to the study protocol and dedicated observer training, including measurement of resting BP as recommended by international guidelines^13^. To gain insights into relationships between specific genes, clinical parameters and the cardiovascular phenotype of the 4 C patients, we conducted correlation analyses with the last set of vascular measurements performed and with calculated progression of these measurements from baseline data at the time of enrollment.

Two-dimensional echocardiography images were obtained for the analysis of left ventricular (LV) volumes and left ventricular mass (LVM), which was calculated according to the Devereux formula^14^ and indexed to height 2.7 (LVMI). The sex- and age-specific LVMI partition values of Khoury *et al*. were applied to define LV hypertrophy^15^. All ABPM measurements were performed using the same portable device type at all sites (Spacelabs 90207–2Q) as described previously^16^. The carotid intima media thickness (cIMT) was obtained either by five averaged measurements on each side or semi-automatically using a portable ultrasound device (Acuson P50, Siemens) with integrated digital image evaluation software (Syngo US Workplace, Siemens Medical Solutions, USA Inc.); since cIMT in children changes with growth, reference values normalized for height and age were established in 1,155 healthy children aged 6–18 years^17^. The central pulse wave velocity (PWV) was measured with the oscillometric Vicorder device^18^, and compared to reference values normalized for height and age were established in a large European pediatric population (1003 healthy children aged 6–18 years)^19^. Data of BP, ABPM and vascular measurements was also expressed as standard deviation scores (SDS) indexed to gender and age or height^8^.

### Statistics

Patient characteristics are provided as mean ± standard deviation or frequencies depending on the scale level of the data. Transcription levels and their ratios were analyzed for each individual gene by multiple two-sample t tests. Statistical significances with correction for multiple comparisons were determined by the Holm-Sidak method, with alpha = 5.0%. The Kruskal-Wallis test with Dunn’s post hoc multiple comparisons test was used to determine differences between calcium contents of the biopsy sample populations. The unpaired t-test with Welch’s correction was performed to analyze differences between IMT-values of biopsies. Spearman correlations were applied to analyze linear correlations between calcium contents and IMT values and correlations between clinical parameters and levels of gene expression in arterial biopsies. All statistical calculations were performed with the GraphPad Prism-Software 6.01 (GraphPad Software, Inc., La Jolla, CA, USA).

The datasets generated during and/or analysed during the current study are available from the corresponding author on reasonable request

## Results

### Patients

A total of 55 arterial biopsies from 4 C study patients were received from participating centres. Due to strict quality control measures including a non-interrupted cooling chain of cryoconserved probes and sufficient material stored in RNAlater, only 26 probes were suitable for IMT and calcium measurements, of which only 15 probes were suitable for PCR measurements.

These 15 children (10 male, 5 female) were 12.0 ± 3.4 years old and had an estimated glomerular filtration rate (eGFR) of 9.4 ± 3.2 ml/min/1.73 m^2^ at the time of biopsy. Serum levels of calcium (2.2 ± 0.2 mmol/l), phosphorus (1.7 ± 0.2 mmol/l) and parathormone (33.5 ± 23.5 pmol/l) were in the normal range. A variety of renal diseases (Supplementary Table S2**)** had been diagnosed 3.6 ± 3.6 years prior to the time of biopsy. The biopsies were obtained from the inferior epigastric artery (n = 9, receiving a kidney transplant), omental artery (n = 4, starting peritoneal dialysis), and radial artery (n = 2).

For pediatric controls, we identified a total of 37 biopsies of aortic tissue from children collected during surgical repair of congenital defects of the aorta or aortic valve. After excluding children younger than 1 year or with cyanotic or hypertensive heart disease or genetic disorders affecting the aortic arch, 20 biopsies were suitable to be used as controls. These biopsies were from 12 boys and 8 girls aged 3.9 ± 4.6 years.

Adult patients with coronary artery disease served as adult controls. A total of 11 biopsies of the internal thoracic artery, collected from non-diabetic patients (7 male, 4 female) aged 55 ± 9 years, were selected for comparison with the pediatric patients.

### IMT and Calcium content of biopsies

Biopsies analyzed for IMT and calcium contents were from 26 children aged 12.3 ± 3.3 years. They had CKD diagnosed 2.9 ± 3.1 years prior to study. The eGFR was 9.1 ± 3.5 ml/min/1.73 m^2^. The biopsies were obtained from the inferior epigastric artery (n = 18, receiving a kidney transplant), omental artery (n = 4, starting peritoneal dialysis), and radial artery (n = 4, starting hemodialysis).

**Figure 1:**
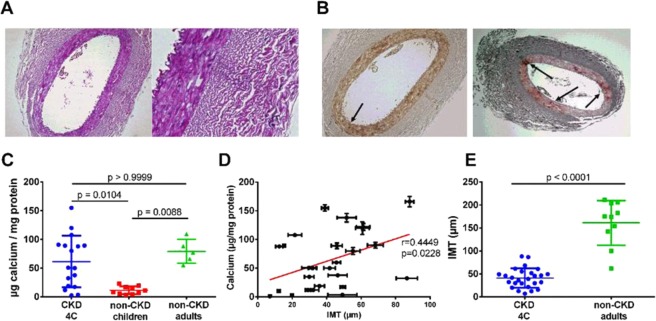
(**A**) H/E-stained arterial biopsy, CKD patient (female, age 13.7 years). Magnification: 10 × (left), 40 × (right) (**B**) V*on Kossa*- (left) and alizarin red-staining (right) in adjacent slices of same biopsy. Von Kossa-stained calcified areas appear black while alizarin-red stained, areas appear dark-red (see arrows). Magnification: 10 × . (**C**) Calcium content of arterial vessel walls as determined by the OCPC method (Kruskall-Wallis test; global p-value: p = 0.0025). (**D**) Linear regression of vessel IMT with the corresponding OCPC-calcium contents in children with CKD (Spearman correlation coefficient). (**E**) IMT of biopsied vessels of children with CKD (4 C Study) and of adult non-CKD patients undergoing coronary bypass operation (Mann-Whitney test). Data are expressed as means ± SD.

The pediatric CKD biopsies showed no atherosclerotic lesions (Fig. 1A,B). The mean IMT of the biopsied vessels was 41.00 ± 21.19 µm, measuring about 25% of the IMT of adult control biopsies (161.00 ± 48.64 µm; Fig. 1C). Only incipient calcifications of the vessel walls were observed, and no overt calcifications were detectable by *von Kossa staining* while alizarin red–stained sections exhibited small areas of mineralized matrix representing 2.5 ± 2.6% of the whole vessel area.

The mean calcium content of the 4 C study biopsies was 63.00 ± 49.94 µg calcium/mg protein and thus, well within the range of the control biopsies from adults (79.26 ± 20.97 µg calcium/mg protein). In contrast, the control biopsies from non-CKD children contained only 11.49 ± 6.87 µg calcium/mg protein. Thus, the calcium content of biopsies from children with CKD was increased ~5-fold compared to biopsies of children with normal kidney function (Fig. 1C).

The calcium contents of biopsies from CKD children correlated weakly with the respective IMT (Fig. 1D). Of note, in contrast to the calcium contents, the IMT of the 4 C biopsies were significantly lower than the respective values of the adult controls (Fig. 1E).

### Gene expression in the 4 C biopsies

#### Gene expressions relative to housekeeping genes

According to their functionality, the 88 genes included in the customized array were clustered into different groups (Table 1) to facilitate the evaluation of the complex gene expression and correlation data sets. First, we determined whether genes in the 4 C biopsies have a higher or lower level of transcription compared to the mean gene expressions of five housekeeping genes. We found distinctly higher or lower expressions for 50 out of 88 analyzed genes. A distinctly higher level of transcription was observed for 18 genes (36%) and a distinctly lower level of transcription for 32 genes (64%). 34% (17/50) of all these genes encoded for extracellular matrix (ECM) components (Table 2). The gene with the highest level of transcription (>1000-fold compared to the mean gene transcriptions of the housekeeping genes) was matrix gla protein (MGP).

**Table 1:** Genes included in customized PCR array*.

Functional category	Genes
Vascular calcification	SP7, RUNX2, CDKN2A, TGM2, BGLAP, S100A12
Extracellular matrix	CTGF, COL1A2, COL2A1, COL6A2, DDR1, VCAN, ELN, TIMP1, TIMP2, MMP1, MMP2, MMP3, MMP7, MMP8, MMP9, MMP14, ADAMTS7, VNN1, FBN1, HSPG2, ACAN, PPARG, HMGA1, EPHX2, HMGB1, COMP
Bone metabolism	SOST, BMP1, BMP2, BMP3, BMP7
Physiological calcification inhibitors	CA2, ANKH, TRIM24, ADIPOQ, HMOX1, FTL, TNFRSF11B, MGP, AHSG, ENPP1, SPP1
Ion channels	TRPV5, TRPV6, KCNN4, KCNN3, KCNMA1, RYR3, S100G
Endoplasmic reticulum stress	HSPA5, HSP90B1, DDIT3
Apoptosis	CASP3, CASP8, BAX
Wnt signaling	AXIN2, SFRP4, FGF2
NF-ĸB signaling	MYD88, NFKB1, RELA
TGF-β signaling	TGFB1, TGFBR1, TGFBR2, LTBP3, SMAD6
Inflammation	IL10, TNF
NO synthases	NOS2, NOS3, PRKG1, NOS1
Various	FGF23, KL, VDR, AGTR2, ANGPT2, SLC25A15, ALPL, IFNG, AGER, CRP
Housekeeping genes	HPRT1, PGK1, EIF2B1, PPIA, ELF1

*For abbreviations, see Supplemental Material.

**Table 2:** Genes with high and low transcription levels in 4 C study biopsies*.

Rank	Gene	Ratio 4 C vs. housekeeping (MEAN ± SD)	Gene group
**Genes with a high transcription level:**			
1	MGP	1184.2 ± 1241.88	Physiological calcification inhibitors
2	HMGB1	17.09 ± 16.90	Extracellular matrix
3	CTGF	16.51 ± 26.64	Extracellular matrix
4	COL1A2	12.53 ± 8.45	Extracellular matrix
5	HSP90B1	10.33 ± 7.88	Endoplasmic reticulum stress
6	SP7	6.22 ± 11.08	Vascular calcification
7	ADIPOQ	5.30 ± 6.89	Physiological calcification inhibitors
8	FBN1	4.71 ± 5.41	Extracellular matrix
9	TIMP2	4.69 ± 3.97	Extracellular matrix
10	ELN	4.62 ± 6.32	Extracellular matrix
11	COL6A2	3.42 ± 3.20	Extracellular matrix
12	TGM2	3.32 ± 3.90	Extracellular matrix
13	MMP2	2.91 ± 2.70	Extracellular matrix
14	TGFBR2	2.80 ± 3.14	TGF-β signaling
15	DDIT3	2.68 ± 2.02	Endoplasmic reticulum stress
16	FTL	2.43 ± 2.18	Physiological calcification inhibitors
17	KCNMA1	2.43 ± 1.89	Ion channels
18	FGF2	1.81 ± 1.22	Wnt-pathway
**Genes with a low transcription level:**			
1	BMP2	0.06 ± 0.09	Bone metabolism
2	DDR1	0.08 ± 0.08	Extracellular matrix
3	SMAD6	0.08 ± 0.08	TGF-β signaling
4	ACAN	0.10 ± 0.11	Extracellular matrix
5	BMP7	0.12 ± 0.21	Bone metabolism
6	KCNN4	0.13 ± 0.19	Ion channels
7	KCNN3	0.13 ± 0.21	Ion channels
8	ALPL	0.15 ± 0.21	Various
9	HMGA1	0.16 ± 0.21	Extracellular matrix
10	TNF	0.18 ± 0.21	Inflammation
11	IFNG	0.18 ± 0.37	Various
12	VDR	0.20 ± 0.29	Various
13	NOS3	0.20 ± 0.14	NO-synthases
14	RYR3	0.20 ± 0.14	Ion channels
15	KL	0.21 ± 0.29	Various
16	VNN1	0.28 ± 0.49	Extracellular matrix
17	NFKB1	0.31 ± 0.21	NF-κB signaling
18	IL10	0.32 ± 0.38	Inflammation
19	HMOX1	0.33 ± 0.36	Physiological calcification inhibitors
20	BMP3	0.33 ± 0.33	Bone metabolism
21	AXIN2	0.34 ± 0.59	Wnt signaling
22	VCAN	0.37 ± 0.49	Extracellular matrix
23	MMP14	0.40 ± 0.56	Extracellular matrix
25	CASP8	0.41 ± 0.40	Apoptosis
26	TNFRSF11B	0.44 ± 0.60	Physiological calcification inhibitors
27	MMP9	0.44 ± 0.49	Extracellular matrix
28	CASP3	0.44 ± 0.55	Apoptosis
29	BGLAP	0.45 ± 0.58	Vascular calcification
30	CDKN2A	0.45 ± 0.48	Vascular calcification
31	AGER	0.48 ± 0.56	Various
32	ADAMTS7	0.49 ± 0.65	Extracellular matrix

*The data are sorted by the magnitude of the ratio of gene expression. Transcription levels and their ratios were analyzed for each individual gene by comparing 4 C and housekeeping genes.

#### Gene expressions relative to pediatric controls

We next compared the gene expressions in the 4 C study biopsies with that of control biopsies from children without CKD (Table 3). Most of the significantly regulated genes (38%; 13/34) related to ECM turnover. The ECM components elastin (ELN), collagen type I (COL1A2), versican (VCAN) and the tissue inhibitor of matrix metalloproteinases type 2 (TIMP2) showed markedly downregulated expression Furthermore, genes encoding for key mediators of vascular smooth muscle cell (VSMC)-mediated vascular calcification such as osterix (SP7), runt-related transcription factor 2 (RUNX2) and the senescence marker cyclin-dependent kinase Inhibitor 2 A (CDKN2A) were highly upregulated, whereas the physiological artery calcification inhibitor ecto-nucleotide pyrophosphatase/phosphodiesterase 1 (ENPP1) was significantly downregulated compared to the healthy control biopsies.

**Table 3:** Significantly altered gene transcription in 4 C study biopsies relative to pediatric controls*.

Rank	Gene	Ratio 4 C vs. non-CKD children(MEAN ± SD)	p-value	Gene-group
**Significantly upregulated genes**				
1	SP7	247.55 ± 644.48	0.0171	Vascular calcification
2	MMP7	70.81 ± 174.42	0.0174	Extracellular matrix
3	NOS2	35.83 ± 97.95	< 0.0001	NO-Synthases
4	RUNX2	24.76 ± 67.45	0.0017	Vascular calcification
5	VNN1	23.89 ± 62.64	0.0394	Extracellular matrix
6	CDKN2A	15.23 ± 25.45	0.0007	Vascular calcification
7	COL2A1	15.20 ± 37.29	0.0146	Extracellular matrix
8	IL10	11.33 ± 27.48	0.0037	Inflammation
9	AGTR2	10.87 ± 33.75	0.0211	Various
10	TNF	8.29 ± 19.54	0.0033	Inflammation
11	BMP3	7.91 ± 19.94	0.0151	Bone metabolism
12	TRPV6	7.66 ± 18.55	0.0024	Ion channels
13	KCNN3	6.60 ± 17.93	0.0281	Ion channels
14	PPARG	5.99 ± 17.96	0.0025	Extracellular matrix
15	MGP	5.20 ± 10.18	0.0018	Physiological calcification inhibitors
16	KCNN4	4.96 ± 12.12	0.0303	Ion channels
17	S100A12	4.84 ± 16.05	0.0023	Vascular calcification
18	HMGB1	4.73 ± 5.78	0.0011	Extracellular matrix
19	CA2	3.61 ± 7.53	0.0125	Physiological calcification inhibitors
20	HMGA1	3.58 ± 7.10	0.0154	Extracellular matrix
21	KL	3.40 ± 6.65	0.0252	Various
22	FGF2	3.09 ± 3.16	0.0001	Wnt-pathway
23	DDIT3	1.66 ± 2.20	0.0491	Endoplasmic reticulum stress
**Significantly downregulated genes**				
1	ELN	0.01 ± 0.04	0.0088	Extracellular matrix
2	COL1A2	0.07 ± 0.11	< 0.0001	Extracellular matrix
3	VCAN	0.18 ± 0.38	0.0065	Extracellular matrix
4	TIMP2	0.27 ± 0.30	< 0.0001	Extracellular matrix
5	LTBP3	0.30 ± 0.62	0.0091	TGF-β signaling
6	ENPP1	0.31 ± 0.72	0.0064	Physiological calcification inhibitors
7	TIMP1	0.42 ± 0.79	0.0085	Extracellular matrix
8	COL6A2	0.45 ± 0.87	0.0405	Extracellular matrix
9	MMP2	0.47 ± 0.66	0.0048	Extracellular matrix
10	BMP1	0.49 ± 0.82	0.0401	Bone metabolism
11	HSP90B1	0.58 ± 0.68	0.0061	Endoplasmic reticulum stress

*The data are sorted by the magnitude of the ratio of gene expression. Transcription levels and their ratios were analyzed for each individual gene by multiple two-sample t- tests comparing 4 C and non-CKD children. Statistical significances with correction for multiple comparisons were determined by the Holm-Sidak method, with alpha = 5.0%.

#### Pediatric CKD vs. adult non-CKD biopsies

We next analyzed differences in gene expression between pediatric CKD biopsies and control biopsies from adults with classical atherosclerosis but normal kidney function. Transcription levels of 21 genes were significantly different between both groups. Thirteen out of these 21 genes (62%) were up- or downregulated in accordance with the gene expression ratios of pediatric CKD biopsies relative to pediatric controls (flagged green in Table 4). Furthermore, one third of the significantly regulated genes (7/21) were involved in ECM regulation. Of note the ratio of ADIPOQ, encoding for adiponectin, was 38. This indicates an upregulation of this adipokine which is considered to function as a physiological inhibitor of arterial calcification^20^.

**Table 4:** Significantly altered gene transcription in 4 C study biopsies relative to adult non-CKD controls*.

Rank	Gene	Ratio 4 C vs. adult controls (MEAN ± SD)	p-value	Gene-group	Flag
**Significantly upregulated genes**					
1	ADIPOQ	37.89 ± 149.38	0.0498	Physiological calcification inhibitors	≠
2	NOS2	25.63 ± 66.88	0.0129	NO-Synthases	✓
3	MMP9	21.89 ± 68.26	0.0438	Extracellular matrix	≠
4	RUNX2	17.36 ± 47.56	0.0279	Vascular calcification	✓
5	TRPV6	16.75 ± 46.19	0.0200	Ion channels	✓
6	IL10	16.08 ± 51.00	0.0440	Inflammation	✓
7	PPARG	15.34 ± 48.09	0.0114	Extracellular matrix	✓
8	S100A12	11.59 ± 37.29	0.0051	Vascular calcification	✓
9	BMP3	6.54 ± 19.62	0.0338	Bone metabolism	✓
10	HMOX1	5.58 ± 10.71	0.0312	Physiological calcification inhibitors	≠
11	MGP	3.76 ± 6.02	0.0309	Physiological calcification inhibitors	✓
12	HMGB1	2.93 ± 4.05	0.0491	Extracellular matrix	✓
13	FGF2	2.88 ± 3.85	0.0092	Wnt signaling	✓
**Significantly downregulated genes**					
1	TIMP2	0.19 ± 0.34	0.0263	Extracellular matrix	✓
2	KCNMA1	0.24 ± 0.30	0.0005	Ion channels	≠
3	KCNMA1	0.24 ± 0.30	0.0005	Ion channels	≠
4	SFRP4	0.25 ± 0.37	0.0030	Wnt signaling	≠
5	TIMP1	0.37 ± 0.78	0.0294	Extracellular matrix	✓
6	RYR3	0.37 ± 0.51	0.0362	Ion channels	≠
7	FTL	0.39 ± 0.63	0.0093	Extracellular matrix	≠
8	COL1A2	0.40 ± 0.60	0.0130	Extracellular matrix	✓

*The data are sorted by the magnitude of the ratio of gene expression Ratios (CKD children vs. adult controls) were compared by two-sample t-tests using the Holm-Sidak method for multiplicity correction with alpha = 5.0%. Multiplicity adjusted p-values are reported. Flags indicate similarities (✓) and non-similarities (≠) in the direction of regulation. For example, NOS2 is significantly upregulated in 4 C patients relative to pediatric and adult controls (by a factor of 35 and 25, respectively). In contrast, SP7 is significantly upregulated relative to pediatric controls (247.55 ± 644.48; p = 0.0171) but not relative to adult controls (24.90 ± 107.06; p = 0.1046; not shown).

#### Adult vs. pediatric controls

To investigate physiological age-related changes in gene expression, we compared the gene expression levels (which were normalized to the five housekeeping genes) between the pediatric and adult non-CKD controls. Differences were found particularly in the ECM gene cluster (Supplementary Table S3). Also, the pro-calcifying transglutaminase was much less expressed in pediatric controls compared to the adult biopsies. However, 70% of all analyzed genes (62/88) were not expressed significantly different between both control groups.

### Comparison of human gene expression data with expression data from animal studies

To gain insights into similarities in CKD induced arterial gene expressions across species, we compared the expression data of the 4 C study biopsies with that obtained from rats after subtotal (5/6) nephrectomy (Table 5**)**.

**Table 5:** Comparison of significantly altered gene transcriptions in children with terminal CKD (relative to pediatric controls) and rats with subtotal nephrectomy (relative to sham)*.

Rank	Gene	Flag
**Significantly upregulated genes:**		
1	SP7	✓
2	MMP7	✓
3	NOS2	✓
4	RUNX2	✓
5	VNN1	✓
6	CDKN2A	✓
7	COL2A1	✓
8	IL10	✓
9	AGTR2	✓
10	TNF	✓
11	BMP3	✓
12	TRPV6	✓
13	KCNN3	✓
14	PPARG	✓
15	MGP	≠
16	KCNN4	✓
17	S100A12	
18	HMGB1	
19	CAR2	✓
20	HMGA1	
21	KL	✓
22	FGF2	✓
23	DDIT3	
**Significantly downregulated genes:**		

Rank	Gene	Flag
1	ELN	≠
2	COL1A2	✓
3	VCAN	≠
4	TIMP2	✓
5	LTBP3	≠
6	ENPP1	≠
7	TIMP1	✓
8	COL6A2	≠
9	MMP2	≠
10	BMP1	✓
11	HSP90B1	✓

*Gene transcriptions were compared between up- and downregulated genes regarding similarities (✓) and non-similarities (≠). The data are sorted by the magnitude of the gene expressions. Notably, the gene expressions of DDIT3, S100A12, HMGB1 and HMGA1 were not determined in the rat customized PCR-array.

Altogether, 95% (18/19) of the significantly upregulated genes in humans were also upregulated in CKD rats. Of note, in contrast to the human vessels, the expression of the calcification inhibitor MGP was downregulated in rat aortas. Contrary to the high level of accordance in the upregulated genes, only 50% of the significantly downregulated genes in humans were also found downregulated in rats with CKD.

### Gene expressions in vessel biopsies depending on their calcium content

We further analyzed the gene expressions within the CKD biopsies to test whether differences due different calcium contents (OCPC-method) could be observed. At first, the 4 C study biopsies were divided into groups with more or less than 80 µg calcium/mg protein, an arbitrary cutoff derived from the average calcium concentration in the biopsies from adults without CKD. Five genes were expressed significantly more strongly in probes with higher calcium contents. These were DNA Damage Inducible Transcript 3 (DDIT3), a regulator of endoplasmic reticulum stress with subsequent induction of apoptosis and the pro-apoptotic Bcl-2-associated X protein (BAX). Further, the physiological calcification inhibitor ferritin (FTL) as well as COL6A2 and RELA were more strongly expressed. The respective proteins are involved e.g. in VSMC anchoring in the tunica media (collagen VI) and in NF-κB signaling (RELA/p65) and their upregulation considered protective^21,22^ (Supplementary Fig. S1).

We then performed a correlation analysis between the expression data of all genes with the respective calcium contents as determined by the OCPC method. As shown in Supplementary Table S4, five genes (DDIT3, FTL, HSPG2, MYD88, TIMP2) were significantly correlated with higher calcium contents.

### Association of arterial tissue gene expression and clinical phenotype

We next investigated whether altered gene transcription in pediatric CKD biopsies (compared with pediatric controls; Table 3) correlated with clinical data (complete variables listed in Supplementary Table S5**)**. The resulting correlation coefficients and the respective p values are shown in Table 6. The LVMI of the CKD patients at baseline was negatively correlated with the upregulated genes KCNN3, FGF2 and with the downregulated genes VCAN, TIMP2, ENPP1, LTBP3, MMP2, HSP90B1, and TIMP1, whereas the change in LVMI during follow-up was negatively associated with only one upregulated gene, SP7.

**Table 6:** Significant correlations between significantly altered gene expressions in 4C-biopsies (compared to pediatric controls) and 4 C patient data*.

Rank	Gene	Correlation with	Correlation coefficient	p-value	Gene-group
**Correlations of significantly upregulated genes**					
1	KCNN3	BMI SDS	−0.5627	0.0233	Ion channels
1	IL10	cIMT	0.8120	0.0005	Inflammation
2	TNF	cIMT	0.6703	0.0147	Inflammation
3	CA2	cIMT	0.6328	0.0236	Physiological calcification inhibitors
4	CDKN2A	cIMT	0.6241	0.0254	Vascular calcification
5	FGF2	cIMT	0.6161	0.0288	Wnt-pathway
1	NOS2	cIMT SDS	−0.6490	0.0374	NO-synthases
1	KCNN3	LVMI	−0.6124	0.0152	Ion channels
2	FGF2	LVMI	−0.5455	0.0354	Wnt-pathway
1	COL2A1	PWV SDS	0.6083	0.0124	Extracellular matrix
2	TRPV6	PWV SDS	0.5266	0.0361	Ion channels
1	TNF	slope_cIMT_SDS	0.8398	0.0046	Inflammation
2	IL10	slope_cIMT_SDS	0.8218	0.0066	Inflammation
3	CA2	slope_cIMT_SDS	0.7833	0.0125	Physiological calcification inhibitors
4	HMGA1	slope_cIMT_SDS	0.7030	0.0346	Extracellular matrix
5	VNN1	slope_cIMT_SDS	0.7030	0.0346	Extracellular matrix
6	FGF2	slope_cIMT_SDS	0.6778	0.0448	Wnt-pathway
1	SP7	slope LVMI	−0.5849	0.0457	Vascular calcification
1	NOS2	Uric acid	0.5614	0.0236	NO-synthases
2	SP7	Uric acid	0.4974	0.0499	Vascular calcification
1	DDIT3	cFGF-23	−0.5784	0.0239	Endoplasmatic reticulum stress
1	FGF2	Years since CKD diagnosis	0.6269	0.0093	Wnt-pathway
2	MGP	Years since CKD diagnosis	0.5546	0.0258	Physiological calcification inhibitors
**Correlations of significantly downregulated genes**					
1	ELN	Age	−0.5274	0.0358	Extracellular matrix
1	TIMP2	BMI SDS	0.6833	0.0045	Extracellular matrix
2	VCAN	BMI SDS	−0.5770	0.0193	Extracellular matrix
3	MMP2	BMI SDS	−0.5270	0.0360	Extracellular matrix
1	COL6A2	CAKUT diagnosis	−0.5272	0.0358	Extracellular matrix
2	RELA	CAKUT diagnosis	−0.5142	0.0416	NF-κB signaling
1	RELA	cFGF-23	−0.5763	0.0245	NF-κB signaling
1	ENPP1	cIMT	0.6249	0.0256	Physiological calcification inhibitors
2	VCAN	cIMT	0.5923	0.0362	Extracellular matrix
1	ENPP1	cIMT SDS	−0.8092	0.0026	Physiological calcification inhibitors
1	VCAN	LVMI	−0.7438	0.0015	Extracellular matrix
2	TIMP2	LVMI	−0.6364	0.0021	Extracellular matrix
3	ENPP1	LVMI	−0.6462	0.0093	Physiological calcification inhibitors
4	LTBP3	LVMI	−0.6369	0.0107	TGF-β pathway
5	MMP2	LVMI	−0.6160	0.0145	Extracellular matrix
6	HSP90B1	LVMI	−0.6011	0.0178	Endoplasmic reticulum stress
7	TIMP1	LVMI	−0.5532	0.0324	Extracellular matrix
1	COL1A2	25-OHD-Vitamin D3	0.5085	0.0443	Extracellular matrix
1	VCAN	iPTH	−0.5534	0.0262	Extracellular matrix
1	BMP1	Slope cIMT SDS	0.8513	0.0036	Bone metabolism
2	VCAN	Slope cIMT SDS	0.6908	0.0394	Extracellular matrix
1	COL6A2	Slope mean 24 h MAP_SDS	0.6205	0.0417	Extracellular matrix
1	TIMP1	Slope PWV SDS	0.5767	0.0391	Extracellular matrix

*Data are sorted by the respective strength of the correlations with the clinical parameters.

Abbreviations: BMI SDS: standard deviation score (SDS) of the body mass index; CAKUT: diagnosis of CAKUT (congenital anomaly of the kidneys and urinary tract); cIMT: carotid intima-media thickness; cIMT_SDS: the SDS of cIMT; LVMI: left ventricular mass index; PWV-SDS: standard deviation score of the pulse wave velocity; slope_cIMT_SDS: monthly change in cIMT-SDS; slope mean24MAP_SDS: monthly change in mean 24-hour arterial blood pressure SDS; slope LVMI: change in LVMI; slope_PWV SDS: monthly change in PWV SDS; years_since_ckd_diagnosis: known duration of CKD.

**Figure 2:**
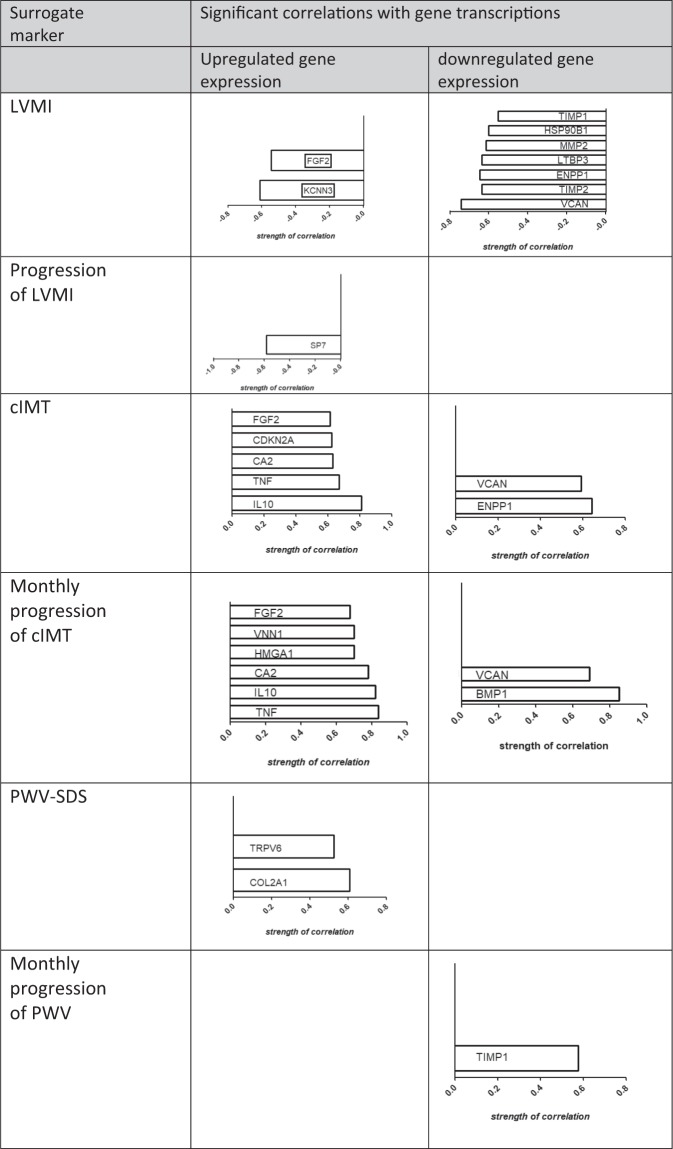
Overview of significantly regulated gene transcripts and their associations with surrogate markers of CVD. Abbreviations: cIMT, Carotid intima media thickness (A. Carotis); LVMI, Left ventricular mass index; PWV, Pulse wave velocity; PWV-SDS, Pulse wave velocity standard deviation score.

The cIMT values of the 4 C patients at baseline showed a significantly positive correlation with the upregulated genes IL10, TNF, CA2, CDKN2A and FGF2 and with the downregulated genes ENPP1 and VCAN. The change in cIMT during follow-up (slope_cIMT_SDS_monthly) showed significant positive correlations with the upregulated genes TNF, IL10, CA2, HMGA1, VNN1 and FGF2 and with the downregulated genes BMP1 and VCAN (Fig. 2).

## Discussion

In this study, arterial biopsies from children with ESKD showed increased calcium contents and significant alterations in gene transcription such as upregulation of calcification inhibitors and extracellular matrix genes. Most observed changes in gene expression were reproducible in the uremic rat model. The changes in arterial gene transcription were frequently associated with cardiovascular phenotypes.

### Calcium accumulation is the dominant finding in incipient remodeling

Arterial tissue calcium levels were increased ~5-fold in the children with ESKD compared to children with normal kidney function and were comparable to adults (age >50) with classical atherosclerosis. The massively increased calcium load went along with an increased IMT of the biopsied arteries, i.e. vascular remodeling.

The gene expression analyses reflected these early changes in the vessels of the 4 C patients. The expressions of DDIT3, FTL, perlecan (HSPG2), MYD88 and TIMP-2 were positively correlated with arterial calcium content. The apoptosis promoting activity of DDIT3^23,24^ may actively contribute to mineralization of the matrix by VSMC^25^. TIMP-2 is a multifunctional protein and has been found positively associated with calcification in radial artery explants of uremic patients^26^. In contrast, FTL (ferritin) and the matrix proteoglycan HSPG2 (perlecan) are considered to have protective function in the vascular wall by inhibiting various pathobiological processes inducing calcification^27,28^. The myeloid differentiation factor MYD88 initiates a pathway resulting in the activation of nuclear factor-kappaB (NF-ĸB) and the production of various proinflammatory mediators. Thus, the correlation of gene transcripts with calcium content in pediatric biopsies indicate the upregulation of pro-calcifying and inflammatory mechanisms but also of calcification inhibitors.

The comparison of gene transcription levels with the pediatric control population further corroborates the upregulation of pro-calcifying factors. The transcription factor SP7 showed the most marked increase in gene expression in the 4 C biopsies (200-fold). This is in line with the central role of SP7 during the transdifferentiation of VSMC to an osteogenic phenotype which is significantly involved in the development of calcifications^25^. The upregulation of MMP-7 (70-fold) and Runx2 (24-fold) are additional indications of the pro-calcifying state.

A further interesting finding was the ~15-fold upregulation of CDKN2A, a regulator of cellular apoptosis and senescence, which is in line with a role for its encoded protein p16INK4a in promoting premature arterial ageing in CKD patients^29^.

Altogether, these data delineate mechanisms of “native” CKD induced accelerated arterial calcification, stiffness and ageing.

### Strong upregulation of calcification inhibitors

Within the 4 C study biopsies we found a very high transcription of MGP, a potent calcification inhibitor^30^, compared to gene expressions of the five housekeeping genes. This is of interest, since previous studies showed an increased MGP expression *in vitro* in response to mineralization^31^. Similarly, the enhanced expression levels of FTL and HSPG2 suggest compensatory protective upregulation. During the onset of calcification, FTL exerts protective effects by its ferroxidase-activity^27^ and HSPG2 is involved in the maintenance of the vessel integrity^32^. In addition, we found a significant upregulation of vascular gene expression of CA2, (carbonic anhydrase 2), HMOX1 (heme oxygenase-1) and ADIPQ (adiponectin), in 4 C study children relative to adult controls. These genes are involved in the prevention of calcification by preventing cellular calcium uptake^33^ and osteoblast transformation of VSMC, respectively^27,34^.

In a previous study of children with ESKD including dialyzed patients, the arterial calcium load was strikingly higher in dialysis compared to pre-dialysis patients^35^. Our findings suggest that the upregulation of inhibitors might protect most pediatric patients from acquiring overt calcifications during the pre-dialysis stage; dialysis–related mechanisms (such as induction of apoptosis) may overcome this protection. In our study, the anti-inflammatory and anti-apoptotic cytokine IL-10 showed the highest level of significance of all variables associated with cIMT and change in cIMT with time in the pediatric ESKD patients. Upregulation of this pluripotent immunoregulatory cytokine (relative to both pediatric and adult controls) could constitute an additional compensatory protective mechanism and one might speculate that similarly to the upregulation of calcification inhibitors, this could be overcome by dialysis – induced inflammatory and apoptotic signals. However, this hypothesis needs confirmation by futher studies.

### Arterial remodeling in CKD starts in the matrix

Modification and reorganization of the ECM is one of the earliest steps in hypertensive vascular remodelling and atherosclerotic cardiovascular disease^36^. Likewise, ECM proteins may be essentially involved in CKD-induced arterial remodeling, as suggested by the fact that about one third of all significantly regulated genes (in biopsies of 4 C study patients relative to pediatric controls) were ECM genes. Of note, ~46% (21/46) of the analyzed transcripts showing significant associations with cardiovascular studies in the pediatric ESKD patients are coding for ECM proteins.

We have previously demonstrated that MMPs play a pivotal role in initiating vascular calcifications in an animal model of CKD^10^. Based on these studies, we expected significant transcriptional changes in MMPs as important contributors to vascular ECM remodeling. However, in the pediatric ESKD biopsies this could only be confirmed for MMP-2 (0.48-fold) while MMP-9 was not significantly regulated compared to the control population. Of interest, MMP-7 expression was highly upregulated in the 4 C study biopsies. In elderly patients with advanced carotid stenosis, high levels of MMP-7 were found in atherosclerotic plaques and considered to promote inflammatory responses and plaque instability^37^. Altogether, one third of the significantly regulated genes in the pediatric patients relative to adult controls code for ECM proteins. This could suggest a more pronounced matrix involvement in incipient CKD-induced arteriopathy compared to established classical atherosclerosis.

### CKD induces similar transcriptional changes in humans and rats

To address an important aspect of preclinical research, we additionally compared the gene expression data from the 4 C biopsies with respective gene expressions in arteries from rats with CKD. The high concordance (95%) in the group of significantly upregulated genes and at least 50% conformity in the group of downregulated genes support the validity of our findings across species and the relevance of experimental rodent models of CKD for the study of CKD associated CVD.

### Association studies confirm the significance of transcriptional changes

We observed some highly significant associations between arterial gene expression and clinical markers of 4 C patients.

The LVMI at baseline, the surrogate marker of CVD showing the greatest increase with each CKD stage in the 4 C study^8^ was significantly associated with the expression levels of several genes in arterial biopsies, indicating their shared involvement in arterial and ventricular remodeling in CKD. However, the change in LVMI during follow-up was only associated (negatively) with the transcription level of a single gene, the transcription factor osterix. Thus, transcription levels in arterial biopsies provided relatively little information regarding the further development of cardiac changes in children with CKD.

In contrast and of particular interest is the observed association of certain tissue transcriptomic changes with the dynamics of carotid IMT thickening documented in the longitudinal clinical study. This parameter indicates the progression of the age-matched cIMT during the prospective observational study. Several significant positive correlations suggest that inflammatory processes (TNF, IL-10), calcification inhibitors (CA2), the Wnt-pathway (FGF-2) and foremost, ECM components (HMGA1, VNN1, VCAN), impact the pathophysiological responses in arteries from children with CKD.

Although standardized systolic blood pressure was the single independent factor significantly associated with all vascular measurements at baseline of the 4 C study^8^ (indicating an extraordinary impact of blood pressure on the cardiovascular phenotype of CKD patients), we could not detect significant associations of blood pressure parameters with arterial gene transcription levels in the present study. A weakly positive correlation of collagen 6A2 gene expression with the change in mean 24-hour arterial blood pressure was the only exception. These findings could indicate that CKD induced vascular remodeling (of peripheral arteries) in the early stage is primarily driven by other factors such as calcium accumulation and matrix rearrangement.

### Limitations

A limitation of the present study is the relatively small number of biopsies included, due to strict quality control of the biopsy material. The study may be further limited by the age differences of the pediatric patient groups and it cannot be ruled out that in the control group, aortic gene expression may have been affected by the presence of the underlying cardiac abnormality, although care was taken in the selection of patients. While it could be argued that aortic tissue (from pediatric controls) is physiologically different from muscular artery tissue (from CKD patients), there is general consensus that CKD-related arterial remodeling is a systemic process affecting conduit and muscular arteries^38^. This notion is further supported by data of this study showing similarities in gene expression changes in human medium-sized arteries and murine aortas after 5/6 nephrectomy, the a widely used animal model for cardiovascular research in CKD. Moreover, customized PCR analysis is a pre-selective, hypothesis-driven approach focusing on proteins known to be involved in vascular remodeling and calcification-associated mechanisms; with this approach we may have missed detection of as yet unknown mechanisms. However, in view of the expected small number of available biopsies, an untargeted approach using a whole genome array would likely have produced a high number of false-negative results and thus, a lower detection rate. It should also be acknowledged that correlations with gene transcription levels indicate associations but not causal relationships.

## Conclusions

CKD-induced arterial disease in children with predialysis ESKD is characterized by calcium accumulation, matrix remodeling, increased stiffness and premature ageing. By measuring arterial gene transcription levels of proteins involved in vascular remodeling, we have characterized molecular mechanisms of CKD-induced arteriopathy at the earliest stage and demonstrated the pathophysiological importance of these genes in relation to the clinical phenotype. Our study provides insights that can serve to initiate further studies of the progression of arterial and cardiac remodeling in patients with CKD.

## Supplementary information

Supplementary Dataset 1

## Data Availability

The datasets generated during and/or analyzed during the current study are available from the corresponding author on reasonable request.
