# Supplementary material for: Arterial tissue transcriptional profiles associate with tissue remodeling and cardiovascular phenotype in children with end-stage kidney disease
Source: Sci Rep. 2019 Jul 16;9:10316. doi: 10.1038/s41598-019-46805-5 (PMC6635360; doi:10.1038/s41598-019-46805-5)
Supplement: Supplementary file 1 — Supplementary Dataset 1 [file 41598_2019_46805_MOESM1_ESM.docx]

**Arterial tissue transcriptional profiles associate with tissue remodeling and cardiovascular phenotype in children with end-stage kidney disease**

Christian Freise, Betti Schaefer, Maria Bartosova, Aysun Bayazit, Ulrike Bauer, Thomas Pickardt, Felix Berger, Lars Melholt Rasmussen; Pia Søndergaard Jensen, Guido Laube, Francesca Mencarelli, Klaus Arbeiter, Rainer Büscher, Sandra Habbig, Kristina Möller, Marietta Kirchner, Franz Schaefer, Claus Peter Schmitt,

and Uwe Querfeld

**Supplementary Table S1**

**Abbreviations and full names of genes**

| **Acronym** | **Full name** | **Acronym** | **Full name** |
| --- | --- | --- | --- |
| ACAN | Aggrecan | CTGF | Connective tissue growth factor |
| ADAMTS7 | A disintegrin and metalloproteinase with thrombospondin motifs-7 | DDIT3 | DNA-Damage-Inducible Transcript 3 |
| ADIPOQ | Adiponectin | DDR1 | Discoidin domain receptor 1 |
| AGER | Advanced Glycosylation End Product-Specific Receptor | EIF2B1 | Eukaryotic Translation Initiation Factor 2B Subunit Alpha |
| AGTR2 | Angiotensin 2 | ELF1 | E74 Like ETS Transcription Factor 1 |
| AHSG | Alpha-2-HS-Glycoprotein / Fetuin A | ELN | Elastin |
| ALPL | Alkaline Phosphatase | ENPP1 | Ecto-nucleotide pyrophosphatase/phosphodiesterase 1 |
| ANGPT2 | Angiopoietin 2 | EPHX2 | Epoxide Hydrolase 2 |
| ANKH | Inorganic Pyrophosphate Transport Regulator | FBN1 | Fibrillin-1 |
| AXIN2 | Axis inhibition protein 2 | FGF2 | Basic Fibroblast Growth Factor |
| BAX | Bcl-2-like protein 4 | FGF23 | Fibroblast Growth Factor 23 |
| BGLAP | Osteocalcin | FTL | Ferritin |
| BMP1 | Bone Morphogenetic Protein 1 | HMGA1 | High-Mobility-Group-Protein HMG-I/HMG-Y |
| BMP2 | Bone Morphogenetic Protein 2 | HMGB1 | High-Mobility-Group-Protein B1 |
| BMP3 | Bone Morphogenetic Protein 3 | HMOX1 | Heme Oxygenase 1 |
| BMP7 | Bone Morphogenetic Protein 7 | HPRT1 | Hypoxanthine Phosphoribosyltransferase 1 |
| CA2 | Carbonic anhydrase-2 | HSP90B1 | Heat Shock Protein 90kDa Beta Member 1 |
| CASP3 | Caspase-3 | HSPA5 | Heat Shock 70kDa Protein 5 |
| CASP8 | Caspase-8 | HSPG2 | Heparan Sulfate Proteoglycan 2 |
| CDKN2A | Cyclin-dependent kinase Inhibitor 2A | IFNG | Interferon gamma |
| COL1A2 | Collagen type I | IL10 | Interleukin-10 |
| COL2A1 | Collagen type II | KCNMA1 | KCa1.1: Calcium-activated potassium-channel (large conductance) |
| COL6A2 | Collagen type VI | KCNN3 | KCa2.3: Calcium-activated potassium-channel (small conductance) |
| COMP | Cartilage Oligomeric Matrix Protein | KCNN4 | KCa3.1: Calcium-activated potassium-channel (intermediate conductance) |
| CRP | C reactive protein | KL | Klotho |
| LTBP3 | Latent-transforming growth factor beta-binding protein 3 | TGFB1 | Transforming growth factor beta-1 |
| MGP | Matrix Gla Protein | TGFBR1 | Transforming growth factor beta receptor 1 |
| MMP1 | Matrix-metalloproteinase-1 | TGFBR2 | Transforming growth factor beta receptor 2 |
| MMP14 | Matrix-metalloproteinase-14 | TGM2 | Transglutaminase-2 |
| MMP2 | Matrix-metalloproteinase-2 | TIMP1 | Tissue inhibitor of matrix-metalloproteinases 1 |
| MMP3 | Matrix-metalloproteinase-3 | TIMP2 | Tissue inhibitor of matrix-metalloproteinases 2 |
| MMP7 | Matrix-metalloproteinase-7 | TNF | Tumor necrosis factor |
| MMP8 | Matrix-metalloproteinase-8 | TNFRSF11B | Tumor Necrosis Factor Receptor Superfamily, Member 11b |
| MMP9 | Matrix-metalloproteinase-9 | TRIM24 | Transcriptional intermediary factor 1α |
| MYD88 | Myeloid differentiation primary response gene 88 | TRPV5 | Transient receptor potential cation channel, subfamily V, member 5 |
| NFKB1 | Nuclear factor NF-kappa-B p105 subunit | TRPV6 | Transient receptor potential cation channel, subfamily V, member 6 |
| NOS1 | Neuronal nitric oxide synthase (nNOS) | VCAN | Versican |
| NOS2 | Inducible nitric oxide synthase (iNOS) | VDR | Vitamin D receptor |
| NOS3 | Endothelial cell nitric oxide synthase (eNOS) | VNN1 | Vanin-1 |
| PGK1 | Phosphoglycerate Kinase 1 |  |  |
| PPARG | Peroxisome proliferator-activated receptor gamma |  |  |
| PPIA | Peptidylprolyl Isomerase A |  |  |
| PRKG1 | cGMP-dependent protein kinase 1 |  |  |
| RELA | Transcription factor p66 |  |  |
| RUNX2 | Runt-related transcription factor 2 |  |  |
| RYR3 | Ryanodine Receptor 3 |  |  |
| S100A12 | S100 calcium binding protein A12 |  |  |
| S100G | Calbindin D9K |  |  |
| SFRP4 | Secreted frizzled-related protein 4 |  |  |
| SLC25A15 | Solute Carrier Family 25 (Mitochondrial Carrier) Member 15 |  |  |
| SMAD6 | SMAD family member 6 |  |  |
| SOST | Sclerostin |  |  |
| SP7 | Osterix |  |  |
| SPP1 | Secreted Phosphoprotein 1 / Osteopontin |  |  |

**Supplementary Table S2**

**Renal diagnoses of patients undergoing arterial biopsy at end-stage CKD***

Congenital anomalies of the kidney and urinary tract (CAKUT) n=13

Cystic kidney diseases n=3

Glomerulonephritis n=2

Purpura Schönlein-Henoch / IgA nephropathy n=2

Alport Syndrome n=1

Congenital nephrotic syndrome n=1

Cystinosis n=1

Familial hypomagnesemia with hypercalciuria and nephrocalcinosis n=1

Hemolytic-uremic syndrome n=1

Ochoa-Syndrome n=1

*26 probes were suitable for IMT and calcium measurements

**Supplementary Table S3**

**Significant differences between absolute gene expressions in biopsies from non‑CKD children relative to non-CKD adult controls**

**Significantly higher gene expressions in non-CKD children**

| Rank | Gene | Ratio:  non-CKD children vs. non-CKD adults  (MEAN±SD) | p-value | Gene-group |
| --- | --- | --- | --- | --- |
| 1 | MMP-2 | 3.94 ± 4.81 | <0.0001 | Extracellular matrix |
| 2 | FBN1 | 3.15 ± 3.60 | 0.0009 | Extracellular matrix |
| 3 | COL1A2 | 5.63 ± 9.00 | 0.0021 | Extracellular matrix |
| 4 | VCAN | 4.30 ± 5.36 | 0.0038 | Extracellular matrix |
| 5 | KL | 3.00 ± 6.00 | 0.0055 | Various |
| 6 | MYD88 | 1.58 ± 1.23 | 0.0127 | NF-κB signaling |
| 7 | ELN | 19.13 ± 66.90 | 0.0247 | Extracellular matrix |
| 8 | COL2A1 | 3.00 ± 8.00 | 0.0386 | Extracellular matrix |

**Significantly higher gene expressions in non-CKD adults**

| Rank | Gene | Ratio:  non-CKD children vs. non-CKD adults  (MEAN±SD) | p-value | Gene-group |
| --- | --- | --- | --- | --- |
| 1 | KCNMA1 | 0.37 ± 0.44 | <0.0001 | Ion channels |
| 2 | HMGB1 | 0.62 ± 0.39 | 0.0006 | Extracellular matrix |
| 3 | FTL | 0.46 ± 0.57 | 0.0047 | Extracellular matrix |
| 4 | TGM2 | 0.15 ± 0.32 | 0.0212 | Vascular calcification |
| 5 | SFRP4 | 0.53 ± 0.83 | 0.0350 | Wnt signaling |
| 6 | TNFRSF11B | 0.44 ± 0.98 | 0.0421 | Extracellular matrix |
| 7 | PRKG1 | 0.45 ± 0.82 | 0.0516 | NO-synthases |
| 8 | NFKB1 | 0.63 ± 0.78 | 0.0591 | NF-κB signaling |

*Ratios (non-CKD children relative to housekeeping genes vs. adult controls relative to housekeeping genes) were compared by multiple t tests using the Holm-Sidak method, with alpha=5.000%.

**Supplementary Table S4**

**Significant correlations of gene expressions with calcium contents of the 4C biopsies (ranked by level of significance)**

| Rank | Gene | Correlation coefficient | p-value | Gene-group |
| --- | --- | --- | --- | --- |
| 1 | DDIT3 | 0.8303 | 0.0029 | Endoplasmic reticulum stress |
| 2 | FTL | 0.7273 | 0.0112 | Physiological calcification inhibitors |
| 3 | HSPG2 | 0.7857 | 0.0208 | Extracellular matrix |
| 4 | MYD88 | 0.7333 | 0.0246 | NF-κB signaling |
| 5 | TIMP2 | 0.6833 | 0.0424 | Extracellular matrix |

**Supplementary Table S5**

**Abbreviations and full names of clinical parameters and surrogate markers correlating with gene expression levels in biopsies of 4C study patients***

| **Vascular parameters** |  | **Other abbreviations** | |
| --- | --- | --- | --- |
| BMI-SDS | Body mass index standard deviation score | Age | Age of patients |
| cIMT | Carotid intima media thickness (A. Carotis) | CAKUT diagnosis | Congenital malformation of kidney and urinary tract |
| cIMT_SDS | Carotid intima media thickness standard-deviation score | cFGF23 | Fibroblast growth factor 23, serum level |
| LVMI | Left ventricular mass index | OHD25 | 25-hydroxyvitamin D, serum level |
| PWV | Pulse wave velocity | PTH | Serum level of parathyroid hormone |
| PWV_SDS | Pulse wave velocity standard deviation score | Uric acid | Serum level of uric acid |
| Slope_cIMT_SDS | Monthly changes over time of IMT_SDS |  |  |
| Slope_PWV_SDS | Change in pulse wave velocity standard deviation score per month adjusted to height |  |  |
| Slope_LVMI | Change in left ventricular mass index per month |  |  |
| Slope_MAP24h_SDS | Change in 24-hour mean arterial pressure standard deviation score per month |  |  |
| Years_since_CKD_diagnosis | Time of observation since CKD diagnosis |  |  |

*Other variables tested (non-significant):

eGFR; estimated glomerular filtration rate

IMT Intima media thickness of biopsies

gender;

Hemoglobin; Hemoglobin serum level (mg/dl)

map24_sds, mean arterial blood pressure standard deviation score;

serum calcium;

serum high density lipoprotein cholesterol;

serum low density lipoprotein cholesterol;

serum phosphorus

syssds; systolic blood pressure standard deviation score;

Slope_eGFR_monthly; monthly change of the eGFR

Slope_map24_monthly, change in mean arterial blood pressure per month slope_syssds_monthly, change in systolic blood pressure standard deviation score per month;

PWV-SDS_a; Pulse wave velocity standard deviation score adjusted for age

PWV_SDS-h,pulse wave velocity standard deviation score adjusted to height

**Supplementary Figure S1**


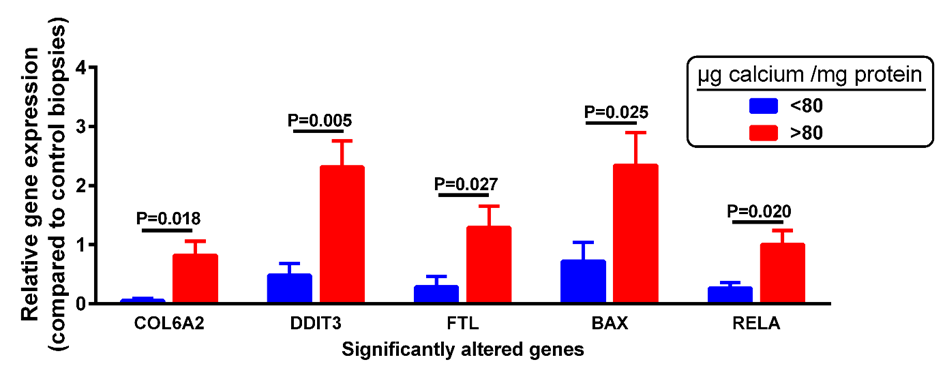


**Supplemental Figure 1.** Significant differences in gene expressions in 4C arterial biopsy specimens dependent on calcium contents. Specimens were grouped according to their calcium content (cutoff 80µg /mg protein).
